# Effort‐Induced Deep Vein Thrombosis of the Right Upper Extremity

**DOI:** 10.1002/ccr3.9680

**Published:** 2024-12-19

**Authors:** Siwar Belhaj Salem, Sahira Shrestha, Jayashree Ravikumar, Linh Huynh, Sara S

**Affiliations:** ^1^ Department of Family Medicine Faculty of Medicine of Monastir Monastir Tunisia; ^2^ Nepal Medical College and Teaching Hospital Kathmandu Nepal; ^3^ Department of Internal Medicine Sri Varadhar Consultation Clinic Chennai India; ^4^ Kansas Health Science Center Wichita Kansas USA; ^5^ Department of Internal Medicine A.R Hospital Ramanathapuram India

**Keywords:** anticoagulants, effort‐induced upper extremity deep vein thrombosis, primary thrombosis, subclavian vein thrombosis, upper extremity

## ABSTRACT

Effort‐induced upper extremity deep vein thrombosis constitutes an uncommon presentation. In this report, we discuss a case of a healthy painter with primary effort‐induced deep vein thrombosis of the upper limb. Such condition occurs often among young individuals who are exposed to vigorous and repetitive movements of retroversion, hyperabduction, and extension of the arm. Following the elimination of all differential diagnoses and secondary causes of UEDVT, duplex venous ultrasound, which is noninvasive, specific, and cost‐effective, is necessary for establishing the diagnosis. Management included early anticoagulation. However, catheter‐directed thrombolysis (CDT) followed by first rib resection (FRR) is by far the most frequently performed therapeutic approach.

## 1 Introduction

Upper extremity deep vein thrombosis (UEDVT) is a condition that comprises about 5% to 10% of all deep vein thrombosis (DVT) cases. UEDVT can be categorized into primary, which is induced by physical effort, and secondary, which frequently occurs as a consequence of medical interventions such as central or peripherally inserted catheters (PICC) and pacemaker insertions [1]. In subclavian and axillary veins, DVT occurs commonly due to central venous catheters, pacemakers, malignancies, or thrombophilia [2]. The European Guidelines encompass a section dedicated to UEDVT, which presents treatment recommendations derived from a comprehensive review of no fewer than 40 distinct references. It is important to acknowledge; however, that the level of evidence supporting these recommendations is categorized as C [3]. Primary UEDVT occurs in 0.5 to 1 per 100,000 people annually and involves thrombosis in the deep veins of the upper extremity due to anatomical anomalies that cause axillary‐subclavian compression [1]. Thrombosis of the subclavian vein was first identified by Sir James Paget [4, 5] in 1875 and later detailed by von Schrötter in 1884, known as Paget–Schroetter syndrome [5]. This syndrome typically affects young, active individuals who experience sudden, severe pain and swelling in the upper extremity following intense upper body exertion [1].

## 2 Case History and Examination

This is a case of a 29‐year‐old previously healthy man presented to the emergency room with a 2‐day history of pain and swelling of the right upper limb. He is a painter by occupation, which involves repetitive movements of abduction, flexion, and external rotation of the right shoulder. Initially the pain was localized at the shoulder and then has progressed to involve the entire limb. He denies any recent trauma. He has no symptoms of chest pain and dyspnea. He does not use tobacco, alcohol, or illicit drugs. He has no family and past history of thrombophilia and malignancy and has no allergies. On examination, the patient is well appearing and is not in acute distress. His vital signs, including blood pressure, heart rate, and pulse oximetry, were in normal range. Examination of right upper extremity revealed palpable tenderness and warm swelling of the homolateral forearm, arm, shoulder, and neck. He had purplish discoloration and dilatation of superficial collateral veins. He had full active range of movements of the right upper extremity with 5/5 muscle strength. Our patient had 2+ symmetric radial and ulnar pulses. Contralateral examination showed normal findings.

## 3 Methods

**FIGURE 1:**
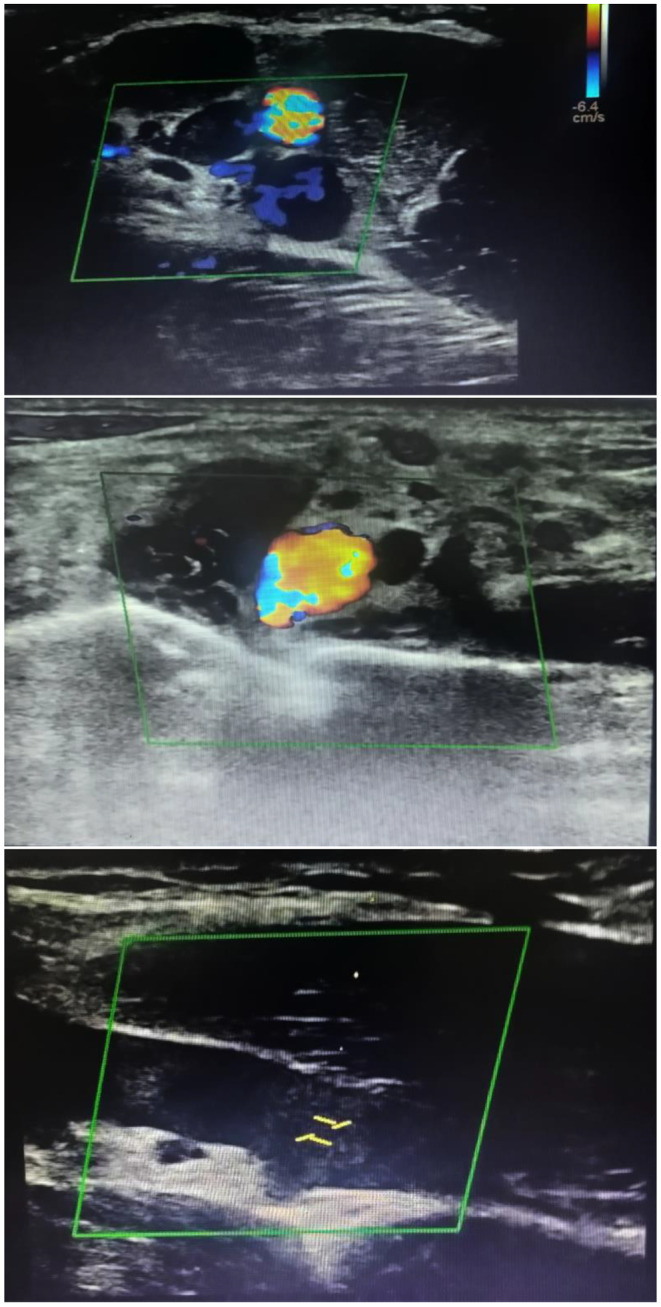
Venous duplex ultrasound of the right upper arm of our patient. The Doppler and B‐mode with absence of compressibility in the right basilic, axillary, and subclavian veins indicating a thrombosis of the studied veins. The pulsed Doppler spectral waveform exhibits an absence of flow augmentation indicating a venous obstruction at the level of the right basilic vein.

An X‐ray of the right shoulder in anterior‐posterior (AP) projection and lateral views were ordered in order to eliminate traumatic injuries and was found to be normal. The second step included imaging with Venous duplex ultrasound that showed an occlusive thrombus in the left basilic vein with extension to the axillary vein and subclavian vein (Figure 1). After eliminating acute complication, mainly pulmonary embolism and phlegmasia cerulea dolens (PCD), the patient was admitted and oral anticoagulation (AC) was initiated. Laboratory and imaging findings were normal (Table 1), excluding thrombophilia and malignancies; the final diagnosis was acute right primary effort‐induced DVT of the right basilic, axillary, and subclavian veins. Upon consultation with hospital's cardiovascular surgery team, the medical treatment was recommended and no surgery was indicated.

**TABLE 1:** Laboratory parameters of the patient.

Test	Result	Normal range	Unit
Urea	3.5	3.3–7.0	mmol/L
Creatinine	60	53–120	μmol/L
Sodium	136	135–145	mmol/L
Potassium	3.9	3.5–4.5	mmol/L
WBC	6000	4500–11,000	cells/μL
MCV	90	80–100	fl
HB	14.2	Males: 13–16 Females: 12–14	g/dL
PLT	180	150–450	10^3^/μL
CRP	5	< 8	mg/L
ESR	10	Males: 0–15 Females: 0–20	mm/h
AST	12	5–45	UI/L
ALT	14	5–45	UI/L
pH	7.37	7.35–7.45	—
HCO3^−^	22	22–28	mmol/L
PCO2	40	35–45	mm Hg
PO2	90	> 80	mm Hg
Anti‐β2 GP1	Negative	Qualitative	Qualitative
ANA	Negative	Qualitative	Qualitative
Ferritinemia	40	Male: 20–250 Female: 10–120	ng/mL
Antithrombin	90	80–120	%
Protein C	85	65–135	IU dL‐1
Protein S	75	60–150	%
Activated protein C resistance ratio	2.7	> 2.1	—
Homocysteine	5.6	< 15	μmol/L

Abbreviations: ALT, alanine transaminase; ANA, antinuclear antibody; Anti‐β2 GP1, antibodies anti‐beta‐2‐glycoprotein‐I; AST, aspartate aminotransferase; CRP, C‐reactive protein; ESR, erythrocyte sedimentation rate; HB, hemoglobin; HCO_3_−, bicarbonates; MCV, mean corpuscular volume; PCO_2_, partial pressure of carbon dioxide; PLQ, platelets count; PO_2_, partial pressure of oxygen; WBC, white blood cells count.

## 4 Conclusion and Results

During the hospitalization, the patients did show improvement, and all symptoms dissipated on the third day. Our patient was then discharged with oral AC for 3 months and was temporary exempted from his job. The patient is now asymptomatic and has returned to his everyday life and pursued his professional activities. Effort‐induced UEDVT is caused by repeated and strenuous physical activity of upper extremity in relatively young healthy adults. Venous ultrasounds in this case confirmed early the diagnosis before the occurrence of fatal complications. Treatment included oral anticoagulants to prevent complications such as pulmonary embolism.

## 5 Discussion

Primary UEDVTs are an uncommon presentation, necessitating a higher level of suspicion from doctors. Patients may be asymptomatic except for limb swelling. This condition typically manifests in the dominant arm of younger athletic individuals engaged in activities that necessitate extensive and repetitive upper extremity motion. Such activities include wrestling, swimming, gymnastics, and activities requiring repetitive ball throwing (i.e., football, baseball, and basketball) or patients who perform strenuous physical labor (e.g., overhead work) [6]. In our case, the patient was a professional painter who experienced significant exposure to repetitive shoulder movements. To the best of the authors' knowledge, this case report is the first relating to painting. Before screening for UEDVT, any probable underlying reasons must be identified. Primary UEDVT may result of thoracic outlet syndrome, upper extremity effort, or are idiopathic. A history of intense activity is the key indicator of effort‐induced UEDVT in otherwise healthy young adults. In contrast, secondary UEDVT is frequently associated with indwelling catheters, malignancy, and pacemakers. DVT mostly affects the subclavian and axillary veins and is frequently associated with malignancy or thrombophilia [7]. Movements of retroversion, hyperabduction, and extension of the arm exert excessive stress upon the subclavian vein, thereby resulting in microtrauma to the endothelial lining and the subsequent activation of the coagulation cascade. Anatomical abnormalities of the thoracic outlet (e.g., cervical rib formation, congenital fibrous bands, hypertrophy of the scalene muscles' tendons, and anomalous insertion of the costoclavicular ligament) may also lead to UEDVT [8]. The symptoms and signs associated with UEDVT exhibit a low specificity, with fewer than 50% of individuals presenting with suggestive symptoms actually have DVT [8]. A venous ultrasound serves as a diagnostic tool. This method is noninvasive and cost‐effective, exhibiting a sensitivity of 87% and a specificity of 85% [9]. This imaging modality reveals dilated veins, thrombotic material, and a lack of venous flow. CT and MRI scans can reveal more anatomical details, but they are more expensive and less accessible. Although magnetic resonance venography has a higher sensitivity, ultrasonography remains the preferred test due to its ease of use and lower cost. Other imaging techniques, such as contrast venography, provide more consistent results but are less often utilized [3, 6, 10]. Venography is invariably performed as an integral component of a multimodal treatment strategy aimed at facilitating catheter‐directed thrombolysis (CDT) and devising a plan for thoracic outlet decompression surgery [8]. D‐dimer tests can help rule out DVT in low‐suspicion instances, though NICE guidelines do not suggest them for diagnosis [6]. The D‐dimer assay has heightened sensitivity of 96%. However, the specificity of D‐dimer testing is relatively low at 47%. Consequently, a positive result necessitates confirmation through a more specific diagnostic procedure, typically an ultrasound [9]. In our case, diagnosis was established with venous ultrasound alone, while the CT scan effectively excluded any anatomical abnormalities. Additionally, our patient did not receive any invasive treatment, and his DVT was managed exclusively through oral AC. The management of our patient aligns with existing literature. Hoexum et al. (2023) reported that primary UEDVT may be treated with AC alone. Notably, following a median follow‐up period of 45 months (with a range of 2–324 months), 54% of patients, who received AC exclusively, reported being symptom‐free. CDT followed by first rib resection (FRR) is the most frequently performed therapeutic approach, demonstrating favorable clinical outcomes in approximately 96% of patients, coupled with a minimal incidence of reported complications. Other therapeutical approaches include CDT + AC or AC + FRR [1].

UEDVTs are routinely treated with AC (low‐molecular‐weight heparin, vitamin K antagonists, or DOACs) for a minimum of 3 months. A consultation with a vascular surgeon is also necessary, and in some cases, CDT is performed. Surgical decompression, such as first rib excision and brachial plexus neurolysis, is the most effective when done within 14 days of diagnosis. Postoperative AC should be continued for 3 months, with a follow‐up contrast venography to ensure vein patency. Noncontact rehabilitation can begin within a month, but contact sports should be avoided until anticoagulant treatment is finished [1, 2, 3, 6, 11]. Complications may manifest in the context of UEDVT, which may include pulmonary embolism, post‐thrombotic syndrome, recurrence of thrombosis, as well as postoperative complications such as pneumothorax, hemorrhagic events, neurological complications, and hemothorax [1]. Taking all factors into consideration, a diverse array of presentations of primary UEDVT are observed, which underscores the extensive variety of variables that influence both its manifestations and therapeutic strategies. Although some similarities exist, such early signs and imaging‐confirmed thrombosis, the distinctive occupational, anatomical, and therapeutic features, managing this condition remains challenging. Consequently, randomized controlled trials are necessary to ascertain the most effective management strategies.

## Author Contributions

**Siwar Belhaj Salem:** conceptualization, data curation, supervision, validation, visualization, writing – original draft, writing – review and editing. **Sahira Shrestha:** formal analysis, investigation, methodology, visualization, writing – review and editing. **Jayashree Ravikumar:** methodology, writing – review and editing. **Linh Huynh:** methodology, writing – review and editing. **Sara S:** investigation, writing – review and editing.

## Consent

A written informed consent was obtained from the patient to publish this report in accordance with the journal's patient consent policy.

## Data Availability

Data sharing not applicable to this article as no datasets were generated or analysed during the current study.
